# The Function of the Thyroid and Parathyroids

**Published:** 1905-03-25

**Authors:** 


### THE FUNCTION OF THE THYROID AND PARATHYROIDS.

It is long since the classical experiments of Sir Victor Horsley established the vital necessity of the internal secretion of the thyroid gland, and the relation of its absence with cretinism and myxœdema. But with the discovery of the parathyroid bodies a revulsion of opinion, such as is not uncommon under similar circumstances, resulted in an extensive belief that these bodies, and not the thyroid itself, were responsible, by their inefficiency, for the phenomena of a-thyroidism. A recent investigation of the subject by Swale Vincent and W. A. Jolly[1] tends to show that neither the thyroid nor the parathyroids are so essential to healthy existence, in laboratory animals at least, as they have been held to be. The parathyroids are usually four in number—two external and two internal. The external ones lie alongside the two lobes of the thyroid gland; the internal ones are buried in the substance of the thyroid lobes. These parathyroid bodies consist of densely-set epithelial cells individually similar to those of the thyroid, but not, as in the gland proper, spaced out to form vesicles.

The authors have experimented with a large number of animals—cats, dogs, foxes, monkeys, rats, and guinea-pigs, and come to the following conclusions:—That the most consistently carnivorous of these animals, the dogs, cats and foxes, commonly die after removal of the thyroid and parathyroids, but not invariably. That monkeys show transient nervous symptoms and generally recover completely, and that rats and guinea-pigs do not suffer at all. It is interesting to note that in none of their experiments were the authors able to produce anything in the nature of myxœdema or cretinism; for though in young animals a stoppage of growth occasionally took place, it was never accompanied by the mental features of cretinism. They found also that when the parathyroids were left after removal of the thyroid, these bodies were capable of developing a vesicular structure similar to that of the normal thyroid, and appeared capable of undertaking the functions of the lost gland.

In view of their experiments these authors hold that myxœdema and cretinism depend upon something more complex than mere thyroid insufficiency. Unfortunately they do not attempt to explain the undeniable benefit resulting from thyroid treatment in the two conditions above-mentioned. For the present, therefore, we can only recognise the experiments as having demonstrated a remarkable physiological distinction between man and the lower animals as regards the metabolic influence of the thyroid secretion, especially remarkable in view of the fact that the gland successfully used for therapeutic purposes in man is one derived from the sheep, which is not only a lower animal, but is herbivorous.

[1] Jour. of Physiology, Dec. 30, 1904.

